# Selective Activation of Human Dendritic Cells by OM-85 through a NF-kB and MAPK Dependent Pathway

**DOI:** 10.1371/journal.pone.0082867

**Published:** 2013-12-30

**Authors:** Carmen Parola, Laura Salogni, Xenia Vaira, Sara Scutera, Paolo Somma, Valentina Salvi, Tiziana Musso, Giuseppe Tabbia, Marco Bardessono, Christian Pasquali, Alberto Mantovani, Silvano Sozzani, Daniela Bosisio

**Affiliations:** 1 Dept. Molecular and Translational Medicine, Università degli Studi di Brescia, Brescia, Italy; 2 Dept. Public Health and Pediatric Sciences, Università degli Studi di Torino, Torino, Italy; 3 Pulmonary Division, Ospedale S. Giovanni Battista, Torino, Italy; 4 Preclinical Research, OM Pharma SA, Meyrin 2/Geneva, Switzerland; 5 Humanitas Clinical and Research Center, Rozzano, Italy; 6 Dept. Biotechnology and Translational Medicine, Università degli Studi di Milano, Milano, Italy; University ofTennessee Health Science Center, United States of America

## Abstract

OM-85 (Broncho-Vaxom®, Broncho-Munal®, Ommunal®, Paxoral®, Vaxoral®), a product made of the water soluble fractions of 21 inactivated bacterial strain patterns responsible for respiratory tract infections, is used for the prevention of recurrent upper respiratory tract infections and acute exacerbations in chronic obstructive pulmonary disease patients. OM-85 is able to potentiate both innate and adaptive immune responses. However, the molecular mechanisms responsible for OM-85 activation are still largely unknown. Purpose of this study was to investigate the impact of OM-85 stimulation on human dendritic cell functions. We show that OM-85 selectively induced NF-kB and MAPK activation in human DC with no detectable action on the interferon regulatory factor (IRF) pathway. As a consequence, chemokines (i.e. CXCL8, CXCL6, CCL3, CCL20, CCL22) and B-cell activating cytokines (i.e. IL-6, BAFF and IL-10) were strongly upregulated. OM-85 also synergized with the action of classical pro-inflammatory stimuli used at suboptimal concentrations. Peripheral blood mononuclear cells from patients with COPD, a pathological condition often associated with altered PRR expression pattern, fully retained the capability to respond to OM-85. These results provide new insights on the molecular mechanisms of OM-85 activation of the immune response and strengthen the rational for its use in clinical settings.

## Introduction

In the last ten years, it has become increasingly clear that dendritic cell (DC) activation is one of the key steps leading to the induction and polarization of the immune and inflammatory response. The innate immune system sense microbes by multiple classes of pattern recognition receptors (PRR), including Toll-like receptors (TLR), NOD-like receptors (NLR), RIG-1-like receptors (RLR) and C-type lectins. As sentinels of the immune system, DC express a vast repertoire of PRR [1]–[3]. Pathogen recognition by PRR activates a transcriptional program leading to the synthesis of molecules required for a rapid, efficient and pathogen-tailored immune-response. For example, PRR sensing extracellular microbes (such as TLR2, TLR5 and some C-type lectins) activate a NF-kB- and MAPK-dependent production of pro-inflammatory cytokines, while PRR recognizing intracellular pathogen-associated molecular patterns (PAMP) (e.g. TLR7-9 and RIG-1) lead to the activation of the interferon regulatory factor (IRF) family of transcription factors and to the production of type I Interferons. Notably TLR4, which senses lipolysaccharide (LPS) as well as other microbial and endogenous danger signals, is known to activate both pathways. Pathogen recognition also induces DC maturation to professional antigen presenting cells, characterized by the expression of co-stimulatory molecules and by a “switch” in the expression of chemotactic receptors that enables them to travel to secondary lymphoid organs, where they meet naïve T cells [4].

OM-85 (Broncho-Vaxom®, Broncho-Munal®, Ommunal®, Paxoral®, Vaxoral®) is the lyophilisate of the water soluble fractions of 21 strain lysates from the following species, which are commonly found in respiratory tract infections: *Haemophilus influenzae, Streptococcus (Diplococcus) pneumoniae, Klebsiella pneumoniae ssp. pneumoniae et ssp. ozaenae, Staphylococcus aureus, Streptococcus pyogenes et sanguinis (viridans), Moraxella (Branhamella/Neisseria) catarrhalis*. Despite OM-85 is used in the prevention of recurrent infections of the airways and acute exacerbations in chronic obstructive pulmonary disease (COPD) [5], [6], little is known about the molecular mechanisms underlying these effects. Bacterial lysates have been empirically, yet extensively, used in the prophylaxis of infections since the beginning of the XX century. The rational for this approach was to stimulate an antibody response against pathogens that may protect mucosal surfaces. According to the current view of DC biology, orally administered bacterial antigen-based drugs may activate mucosal DC by acting as PRR ligands. In turn, DC would induce antigen-specific T lymphocytes contributing to the isotype switch towards IgA via the secretion of cytokines such as transforming growth factor- β (TGF-β) or members of the tumor necrosis factor (TNF) family such as B-cell activating factor (BAFF) and a proliferation-inducing ligand (APRIL) [7]. After maturation, IgA-producing plasmablasts get into the blood stream and preferentially home to mucosal-associated lymphoid tissues of different organs including the airways [7]. Consistent with this view, the protective effect of OM-85 in a model of type I diabetes was found to depend on the presence of TGF-β [8], while human studies indicated that OM-85 increased the content of secretory IgA in bronchoalveolar lavage fluid [9]. In addition, several works indicated that OM-85 may act on DC [8], [10], [11], but the mechanisms underlying this interaction and its biological outcomes have never been investigated thoroughly.

Aim of the present research was to systematically investigate the activation properties of OM-85 on human DC subsets. Here, we report that OM-85 activates human DC via the NF-kB and MAPK pathways. This results in a mild pro-inflammatory activation of DC that release selected cytokines and chemokines, which in turn may favor the recruitment of innate effector cells to epithelial surfaces and promote lymphocyte function. This effect is increased by the presence of suboptimal concentrations of pro-inflammatory stimuli mimicking a pre-existing, subclinical bacterial colonization.

## Materials and Methods

### Ethics statement

The study was approved by the Ethical Committee Intercompany A.O.U San Giovanni Battista and A.O.C.T.O Maria Adelaide (Turin, Italy) and conformed to the principles of the declaration of Helsinki. Blood was taken for routine diagnostic tests and patients provided their written informed consent.

### Cell preparation and culture

Peripheral blood mononuclear cells (PBMC) were isolated from buffy coats of healthy blood donors (through the courtesy of the Centro Trasfusionale, Spedali Civili di Brescia, Brescia, Italy) by Ficoll gradient (Ficoll-Paque™ Plus, GE Healthcare, Israel). Highly enriched blood monocytes were obtained from PBMCs by immunomagnetic separation using anti-CD14-conjugated magnetic microbeads (Miltenyi Biotech, Germany). DC were differentiated from monocytes cultured for 6 days at 8×10^5^/ml in tissue culture plates in RPMI 1640 complemented with 10% FCS, 50 ng/ml GM-CSF (ProSpec Technogene, Israel) and 20 ng/ml IL-4 (ProSpec Technogene). Myeloid (MDC) and plasmacytoid dendritic cells (PDC) were immunomagnetically sorted with anti-blood antigens BDCA-1 and BDCA-4 cell isolation kits respectively (Miltenyi Biotech) [12], [13]. DC maturation was induced by incubation with the indicated concentrations of LPS (*Escherichia coli* 055:B5, Sigma-Aldrich, USA) or OM-85 (OM PHARMA, Switzerland) for 24 hours (1×10^6^ DC/ml). The bacterial endotoxin content of OM-85 was 0.300 EU/ml (0.03 ng/ml) as determined by the producer using the Endosafe®-PTS™, a handheld spectrophotometer that utilizes FDA-licensed disposable cartridges based on the LAL (Limulus Amoebocyte Lysate) method (USP<85>Bacterial Endotoxins Test, chromogenic technique; Eur. Ph. 2.6.14. Bacterial Endotoxins, Method D. Chromogenic kinetic method). Where indicated, cells were stimulated with 1 ng/ml or 10 ng/ml TNF-α and 500 U/ml IFNγ (ProSpec Technogene), PDC were stimulated with 2 µg/ml CpG 2116 (Invivogen, Ca, USA) or OM-85 in the presence of 20 ng/ml IL-3 (ProSpec Technogene).

Polymorphonuclear Neutrophils (PMN) were isolated after Ficoll and 4% Dextran (Sigma Aldrich) gradient separation. To lyse red blood cells, collected neutrophils were treated with 0.2% NaCl (50″) and 1.2% NaCl (Sigma Aldrich). Highly purified blood PMN were obtained by using “Human Neutrophil Enrichment Kit” and “Purple EasySep Magnet” (StemCell Technologies, USA) according to the manufacturer's instructions.

THP-1 (human acute monocytic leukaemia cell line) were purchased from American Type Culture Collection (ATCC, Manassas, VA, USA) and were cultured in RPMI 1640 complemented with 10% FCS.

### Cytoplasmic and nuclear extracts preparation

Cytoplasmic proteins were separated in L1 buffer (50 mM Tris-HCl, pH 8.0; 2 mM EDTA; 0.1% NP-40 and 10%glycerol) with inhibitors (1 mM Na_3_VO_4_, 2 mM DTT, 1 mM NaF, 1 mM PMSF and Protease Inhibitors Cocktail, all reagents were purchased from Sigma-Aldrich). Nuclear pellets were washed twice with L1 buffer with inhibitors and then lysed in NP-40 Lysis buffer (50 mM Tris-HCl, pH 8.0; 250 mM NaCl; 1 mM EDTA; 0.1% NP-40 and 10% glycerol) with inhibitors. Quantification of protein content was performed by Bradford method according to standard protocols.

### Western Blot

Equal amounts of cytoplasmatic or nuclear extracts were boiled for 5 minutes in a loading buffer containing 5% glycerol, 0.001% bromophenol blue, 4.5% SDS and 10% 2-mercaptoethanol. Samples were run through 6–12% polyacrylamide gel and electrophoretically transferred to PDVF membrane (ImmobilionTM, Millipore, Bedford, MA). After blocking of aspecific binding (1% BSA, Sigma-Aldrich), membranes were incubated with the indicated primary antibodies (NF-kB p65, IkBα, ATF2 and c-Jun and β-actin from Santa Cruz Biotechnology, Ca; pERK1/2 from Cell Signalling Technologies, Massachusetts, USA; Lamin A/C from Serotec, UK) for 1 hour at room temperature and then incubated with peroxidase-conjugated secondary antibody (1∶5000; anti-rabbit-HRP; Santa Cruz Biotechnology) for 45 minutes at room temperature. Super Signal® West Pico Chemiluminescent Substrate (Pierce, Rockford, USA) was used to visualize protein bands.

### Electro Mobility Shift Assay (EMSA)

NF-kB DNA binding activity was assessed by EMSA by using the IgG-kB probe (FAM Biotech srl, Brescia, Italy) [14]: GTACGGAGTATCCAGTTGAGGGGACTTTCCCAGGC.

Non-radioactive EMSA was performed using DIG Gel Shift Kit, 2^nd^ Generation (Roche, Mannheim, Germany), according to the manufacturer's instructions. Briefly: single stranded oligonucleotides were annealed and 3′end-labeled with digoxigenin-11-ddUTP through Terminal Transferase. Labeled DNA fragments containing the sequence of interest were incubated with nuclear cell extracts (see above), binding buffer, poly [d(I-C)], poly L-lysine, and/or unlabeled oligonucleotide (125-fold excess) for EMSA competition, and/or specific antibody (1 µg/sample) for supershift assays. Following a 15 minute-incubation at room temperature, the reactions were transferred into a pre-runned 6% native polyacrylamide mini-gel (0.75 mm thick) and submitted to gel electrophoresis (120 V, 1 hour). Following electrophoretic separation, oligonucleotide-protein complexes were transferred by electro-blotting (400 mA, 30 minutes) onto specific positively charged nylon membranes (Roche) and detected using an anti-digoxigenin antibody conjugated with alkaline phosphatase, revealed by autoradiography upon application of a specific luminescent substrate (CSPD, Roche). The generated chemiluminescent signals were recorded on X-ray films.

### Reporter plasmid preparation and luciferase assay

A NF-kB reporter plasmid was prepared for stable transfection. The pGL4.27 [luc2P/minP/Hygro] vector (Promega, USA), was selected as the backbone. A tandem of three NF-kB responsive elements [14] were cloned into the pGL4 vector using the NheI and BglII sites of the multiple cloning region to generate the pGL4-kB reporter plasmid. THP1 transfection was performed using Amaxa® Cell Line Nucleofector® Kit V (Lonza Cologne AG, Germany), according to the manufacturer's instructions. 48 hours later, cells were put into selection with 200 µg/ml of Hygromycin B (Calbiochem, Merck Chemicals Ltd., UK) as assessed by previously performed viability experiments. Luciferase assay was performed using ONE-Glo™ Luciferase Assay System (Promega), according to the manufacturer's instructions. QuantiLum® Recombinant Luciferase (Promega) was used for preparing the luciferase standard curve according to the manufacturer's instructions.

### Chemotaxis

PMNs migration was evaluated using a chemotaxis microchamber technique (NeuroProbe, Pleasanton, CA) using a PVP-free polycarbonate filter (5 µm pore size; NeuroProbe). The chamber was incubated at 37°C for 60 minutes. At the end of the incubation, filters were removed and stained with Diff-Quik (Baxter, Rome, Italy), and 5 high-power oil-immersion fields (magnification 1000×) were counted. All experimetal points were performed in triplicate. DC supernatants were diluted in RPMI to obtain a chemotaxis medium at 1% FCS concentration. For inhibition experiments, 10 mM purified recombinant M3 (a gift from A. Alcami, Centro de Biologia Molecular Severo Ochoa, Madrid, Spain), a broad specificity chemokine-binding protein, was added to the RPMI used to diluted the supernatants, immediately prior of the chemotactic assay. To block G protein-coupled receptors (GCPR) cells were incubated 90 minutes at 37°C with 750 ng/ml of *Bordetella Pertussis* Toxin (Calbiochem, San Diego, CA) and then washed and resuspended in fresh medium before the assay.

### Real-time PCR

RNA was extracted using TRIzol® reagent, according to the manufacturer's instructions. After RNA purification, sample were treated with DnaseI to remove contaminating genomic DNA. Reverse transcription was performed using random hexamers and M-MLV RT. All reagents were purchased from Invitrogen. Gene-specific primers were purchased from FAM Biotech. Sequences are the following: hHPRT forward, 5′-CCAGTCAACAGGGGACATAAA-3′, and hHPRT reverse, 5′-CACAATCAAGACATTCTTTCCAGT-3′; hIL-6 forward, 5′-CACACAGACAGCCACTCACCTC-3′, and hIL-6 reverse, 5′-TCTGCCAGTGCCTCTTTGCTGC-3′; hCXCL8 forward, 5′-AGACAGCAGAGCACACAAGC-3′, and hCXCL8 reverse, 5′-ATGGTTCCTTCCGGTGGT-3′; hIkBα forward, 5′-GTCAAGGAGCTGCAGGAGAT-3′, and hIkBα reverse, 5′-ATGGCCAAGTGCAGGAAC-3′; hTNF-α forward, 5′-CAAGCCTGTAGCCCATGTTGTAG-3′, and hTNF-α reverse, 5′-CCTGGGAGTAGATGAGGTACAGG3′; hCOX-2 forward, 5′-gctttatgctgaagccctatga-3′, and hCOX-2 reverse, 5′-tccaactctgcagacatttcc-3′;’. The iQ™ Sybr Green Supermix (Bio-Rad Laboratories Inc., Hercules, CA, USA) was used to run relative quantitative real-time PCR of the samples according to the manufacturer's instructions. Reactions were run in triplicate on an iCycler™ (Bio-Rad Laboratories Inc.) and generated products analysed with the iCycler™ iQ Optical System Software (Version 3.0a, Bio-Rad Laboratories Inc.). Gene expression was normalized based on HPRT mRNA content. Data are displayed as 2^−ΔΔCt^ values.

### ELISA

Human IL-6, IL-10, IL-12p70, CCL3, CCL20, CCL22, CXCL1, CXCL6, CXCL8 and APRIL protein levels in supernatants were measured by DuoSet® sandwich ELISA according to the manufacturer's instructions (DuoSet® ELISA Development System, R&D System, Minneapolis, USA). Human IFNα and BAFF protein levels were measured by sandwich ELISA from Bender (Austria) and Adipogen (CH) according to the manufacturers' instructions.

### PBMC and MoDC preparation from healthy and COPD patients

A total of seven COPD patients were enrolled while the control group consisted of seven healthy volunteers. Clinical information of patients are listed in Table 1. Peripheral blood mononuclear cells were isolated from whole blood samples of patients and healthy donors by Ficoll gradient. Monocytes and MoDC were prepared as described for buffy coats. 1×10^6^ PBMC or MoDC/ml were stimulated with the indicated concentrations of OM-85 (OM PHARMA) for 24 hours. Where indicated, cells were stimulated with 100 ng/ml TNF-α and 500 U/ml IFNγ. Supernatants were collected and tested by ELISA.

**Table 1:** Characteristic of COPD patients enrolled in the study.

Name	Age	Sex	Age diagnosed COPD	Disease stadium	Therapy	Notes
**P1**	84	M	64	Severe	Inhaled β2 agonist bronchodilatator, inhaled anticholinergics	Chronic respiratory failure (O2 therapy)
**P2**	73	F	57	Moderate/severe	Inhaled β2 agonist bronchodilatator, inhaled anticholinergics	None
**P3**	68	F	55	Severe	Inhaled β2 agonist bronchodilatator, inhaled steroid, inhaled anticholinergics	Chronic respiratory failure (O2 therapy)
**P4**	74	F	60	Very severe	Inhaled β2 agonist bronchodilatator, inhaled steroid, inhaled anticholinergics, frequent administration of courses of antibiotics for chronic bronchial infection	Chronic respiratory failure (O2 therapy)
**P5**	66	M	56	Severe	Inhaled β2 agonist bronchodilatator, inhaled steroid	Smoker
**P6**	56	M	55	Faint	Inhaled anticholinergics	Smoker
**P7**	65	M	64	Very severe	Inhaled β2 agonist bronchodilatator, inhaled steroid, inhaled anticholinergics	None

Table reports the main features (age, sex, age at diagnosis of COPD, disease stadium, therapy and notes) of selected patients **(P).**

### Statistical analysis

Comparisons among treatments were performed by Student's *t*-test, by Dunnett's Multiple Comparison Test or by analysis of variance, as appropriate and as indicated in figure legends.

## Results

### Activation of the NF-kB and MAPK pathways by OM-85 in human monocyte derived DC (MoDC)

**Figure 1:**
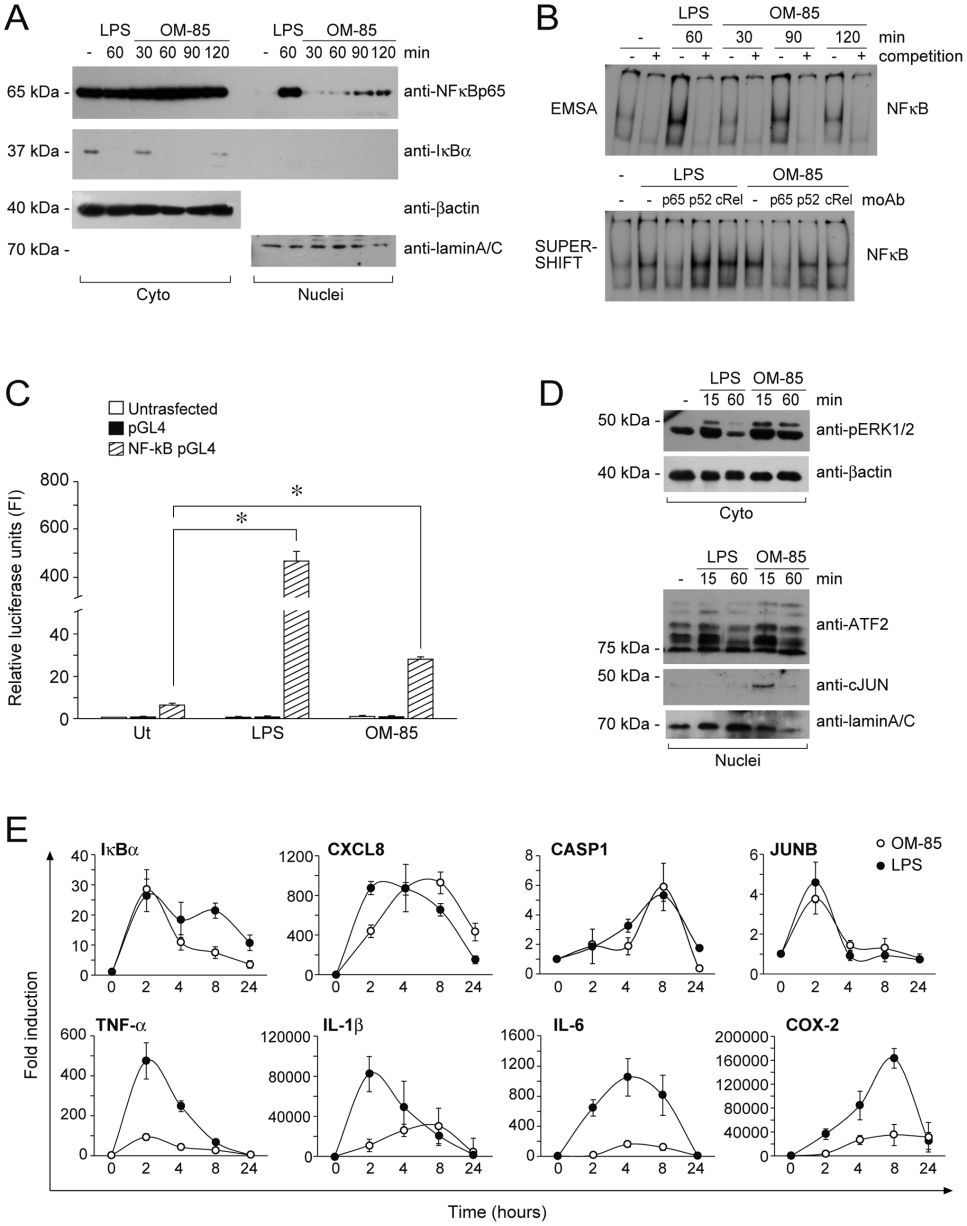
Activation of the NF-kB and MAPK pathways by OM-85 in MoDC. **A**) Immature human MoDC were stimulated with 100 µg/ml OM-85 for 30, 60, 90 and 120 minutes. 100 ng/ml LPS was used as a positive control. After cell lysis and protein fractionation, cytoplasmic (Cyto) and nuclear (Nuclei) extracts were blotted against NF-kB p65 and IkBα. β-actin and Lamin A/C represent loading controls for cytoplasmic and nuclear proteins respectively. The image depicts results obtained in one representative donor out of eight. **B**) EMSA (upper panel) and supershift (lower panel) showing the induction of NFkBp65-DNA binding activity by OM-85 in human moDC stimulated as in A). Signal specificity was assessed by competing each sample with a 125-fold excess unlabeled probe (lanes 2,4,6,8,10 upper panel). The image depicts results obtained in one representative donor out of four. **C**) OM-85 induces the production of luciferase in THP1 cells bearing a NF-kB-reporter plasmid (NF-kB pGL4, striped histograms). THP1 cells were stimulated with 1 µg/ml LPS and 1000 µg/ml OM-85. As expected, THP1 untransfected cells (untransfected, empty histograms) did not produce luciferase in response to stimulation. Similar results were obtained when cells were transfected with the pGL4 empty backbone (pGL4, black histograms). Results are expressed as mean+/−SD of three independent experiments. *P value<0.01 by Dunnett's Multiple Comparison Test. **D**) OM-85 activates the MAPK pathway. Cell extracts prepared as in A) were blotted with antibodies specific for phophorilated ERK1/2 (Cyto, upper panel) and total ATF2 and c-Jun (Nuclei, lower panel). β-actin and Lamin A/C represent loading controls for cytoplasmic and nuclear proteins respectively. The image depicts results obtained in one representative donor out of three. **E**) OM-85 induces NF-kB- and MAPK-dependent gene transcription. Immature MoDC were stimulated with 100 µg/ml OM-85 (open circles) and 100 ng/ml LPS (black circles) for 2, 4, 8 and 24 hours. After RNA extraction, reverse transcription and DNAse I digestion, samples were amplified by Q-PCR using gene-specific primers. Results represent means+/−SE of three independent donors and are expressed as fold induction (FI) over unstimulated samples (0).

Triggering of most of the known PRR activates the cascade leading to NF-kB nuclear translocation and transactivating activity. In order to test the capability of OM-85 to induce NF-kB nuclear translocation in human MoDC, we performed cell fractionation and western blot analysis (Figure 1). As expected, LPS induced IkBα degradation (Figure 1A, center panel) and p65 nuclear translocation at 60 minutes (upper panel). Interestingly, IkBα degradation and p65 nuclear translocation could also be observed when cells were stimulated with OM-85 although with a slower kinetic and in a weaker manner. OM-85 also induced NF-kB DNA-binding activity with a kinetic similar to that observed in western blot experiments (Figure 1B, upper panel). Supershift experiments (Figure 1B, lower panel) demonstrated that p65 is the NF-kB family member mostly involved in MoDC activation by OM-85. These findings are further supported by the evidence that OM-85 induced significant luciferase expression by a reporter NF-kB plasmid stably transfected in THP1 cells, although being less potent than LPS (Figure 1C). Since the signaling pathways that activate NF-kB also activate MAPK, the effects of OM-85 was also investigated on key events of the MAPK cascade. Figure 1D shows that OM-85 induced both ERK1/2 phosphorylation and nuclear translocation of activating transcription factor-2 (ATF2) and c-Jun, two members of the activating protein-1 (AP-1) family of transcription factors. On the contrary, no p38 phosphorylation could be detected in these experimental conditions (not shown).

The activation of these two pathways was also investigated by real time PCR. Figure 1E shows that OM-85 induced the accumulation of MAPK and/or NF-kB target gene mRNA. In some cases, such as for IkBα, CXCL8, caspase 1 and JUNB (upper row), the induction was similar to that observed with LPS, while other genes (such as TNF-a, IL1β, IL-6 and Cyclooxygenase-2, lower row) were induced in a weaker manner.

Of interest, we found that OM-85 did not activate the IRF pathway since no nuclear translocation of IRF3 and IRF7 could be detected by western blot, nor DNA-binding activity by EMSA (Figure S1A and B). These negative results were reinforced by no induction of known IRF target genes, such as IRF1, IRF3, IRF5, IRF7, CXCL9, CXCL10 and IFNβ1 (Figure S1C and not shown). Also, no secretion of the IFNβ1 protein could be detected by ELISA (Figure S1D).

### OM-85 induces B cell activating cytokines and promotes PMN recruitment

**Figure 2:**
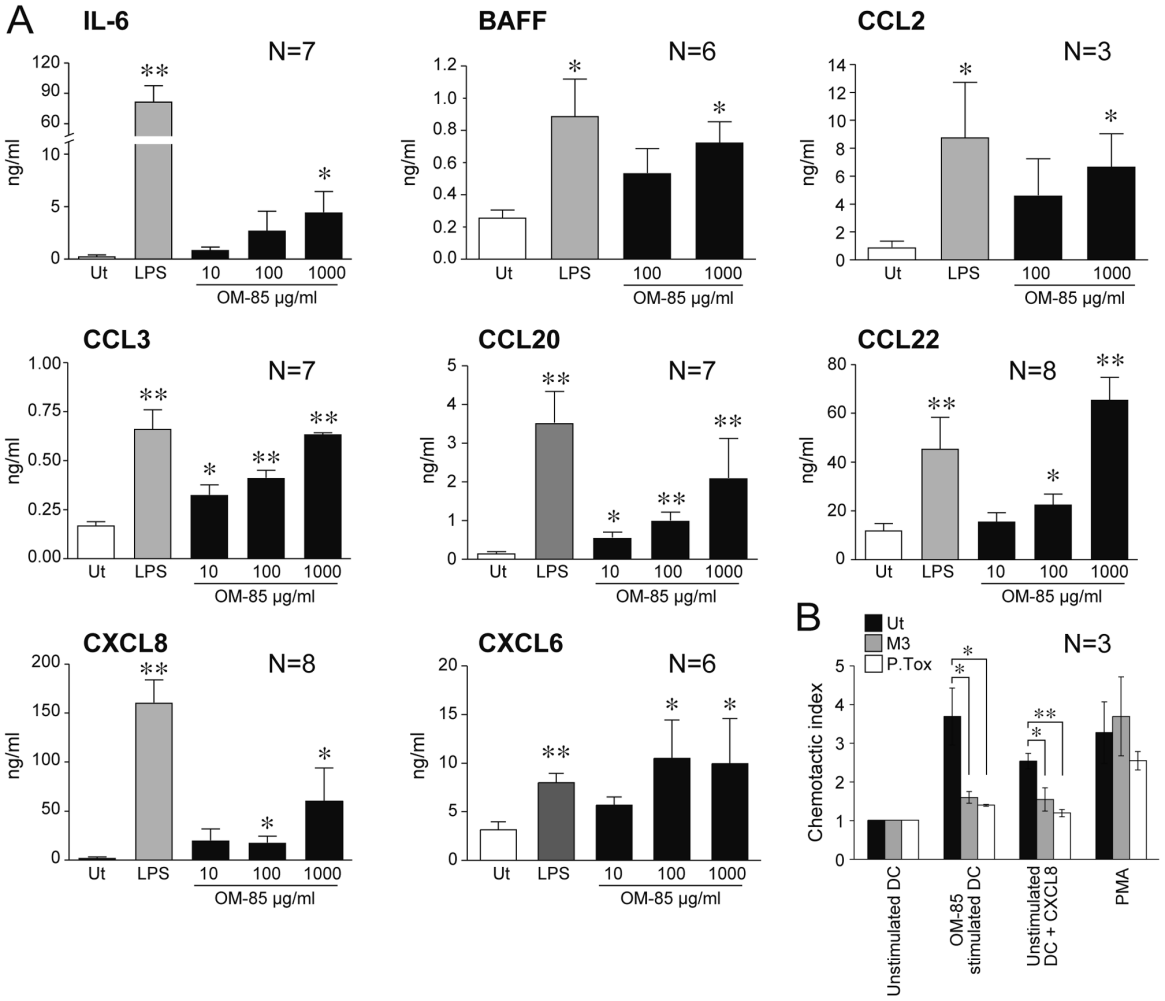
Induction of selected cytokines and chemokines by OM-85 in MoDC. **A**) MoDC (10^6^/ml) were stimulated with OM-85 as indicated and with 100 ng/ml LPS (IL-6, BAFF, CCL2, CXCL8 and CXCL6) or 10 ng/ml LPS (CCL3, CCL20 and CCL22) as a positive control. After 24 hours, supernatants were collected and analyzed by ELISA. Ut = untreated. *P<0.05 and **P<0.01 by Dunnett's Multiple Comparison Test. **B**) Supernatants of MoDC stimulated with OM-85 induce a G-protein-dependent migration of PMN. As a comparison, migration of PMN was elicited with unstimulated supernatants+CXCL8 and PMA. As expected, migration to CXCL8 was inhibited by both 10 nM M3 and 750 ng/ml, *Pertussis toxin* (P.Tox) while migration to PMA was not. Results are expressed as chemotactic index over migration to supernatants of unstimulated MoDC and represent means+/−SD of three independent Boyden chamber experiments. *P value<0.05 and ** P value<0.01 by Dunnett's Multiple Comparison Test.

Activated DC shape the immune response through the secretion of pro- and anti-inflammatory cytokines. Thus, the ability of OM-85 to regulate cytokine and chemokine production was investigated in MoDC (Figure 2). Among the cytokines investigated, OM-85 induced the secretion of IL-6 and BAFF. In addition, OM-85 induced the release of the chemokines CCL2, CCL3, CCL20 and CCL22 in a dose-dependent manner and with a strength comparable to LPS (Figure 2A). Interestingly, OM-85 potently stimulated the production of the PMN-attracting chemokines CXCL8 , CXCL6 and CXCL1 (Figure 2A and not shown). Boyden chamber experiments showed that supernatants of OM-85-stimulated MoDC were active in inducing the migration of freshly purified blood PMN and that this activity was inhibited by cell pretreatment with *Bordetella pertussis* toxin, an inhibitor of GαI protein, and M3, a viral chemokine binding protein [15] (Figure 2B); OM-85 per se was not chemotactic for PMN (not shown). In our experimental conditions, OM-85 did not significantly enhance the secretion of other pro-inflammatory, antiviral and chemotactic mediators, such as TNF-α, IL-1β, IFNβ1, IL-12, IL-23, APRIL, CCL4, CCL19 and CXCL10 (Figure S1D and not shown).

### Synergistic interaction of OM-85 and pro-inflammatory agonists for cytokine production

**Figure 3:**
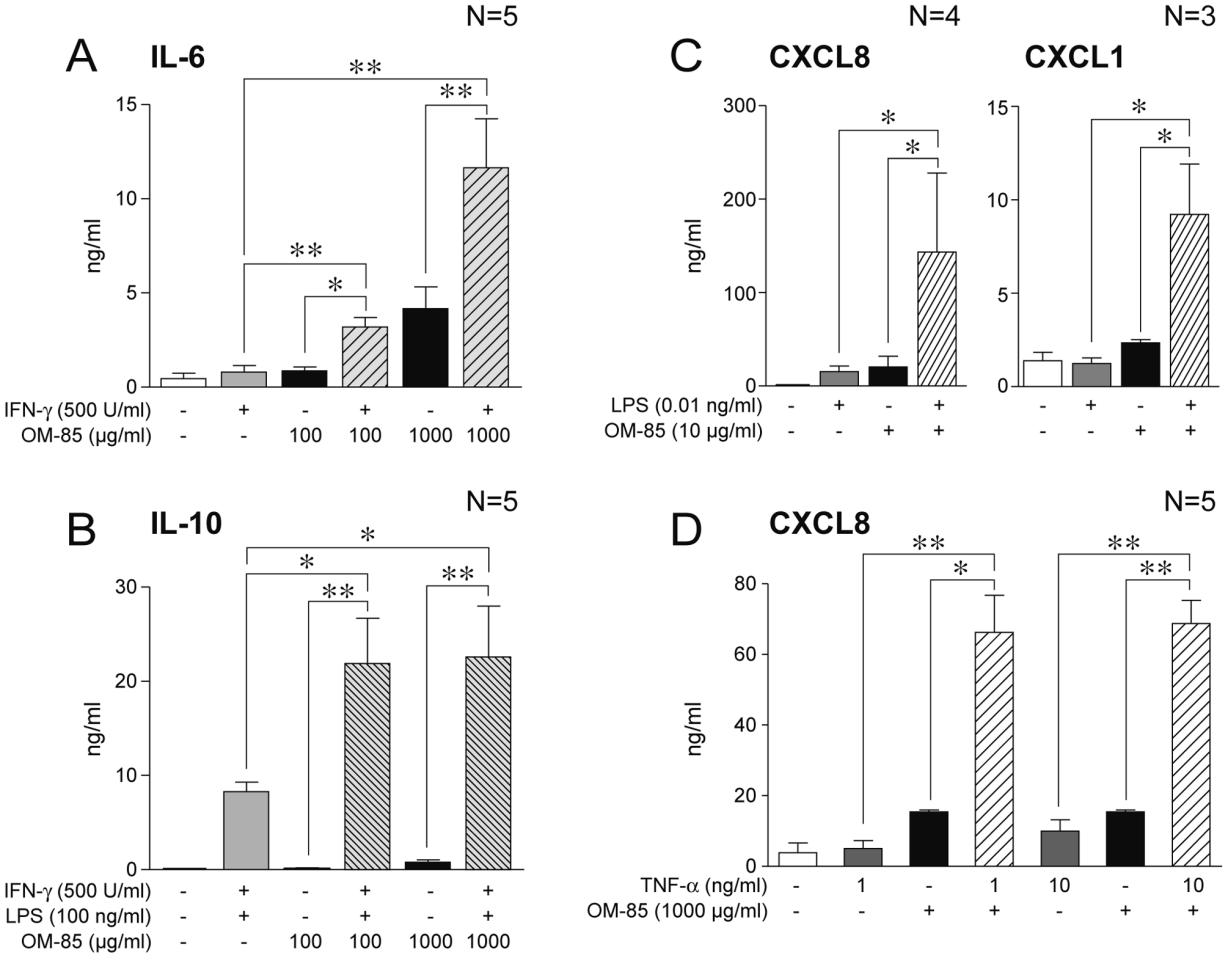
Synergism between OM-85 and pro-inflammatory agonists in MoDC. MoDC (10^6^/ml) were stimulated as indicated. After 24 hours, supernatants were collected and analyzed by ELISA. *P<0.05 and **P<0.005 by paired Student's *t* test.

The ability of OM-85 to induce cytokine production in the presence of pro-inflammatory or immuno-activating stimuli was then investigated. Figure 3A shows that the induction of IL-6 is potently enhanced (3 to 4-fold) in the presence of inactive IFNγ concentrations. Similarly, OM-85 induced a three-fold increase in the IFNγ/LPS-induced release of IL-10, an anti-inflammatory, B cell activating cytokine (Figure 3B).

In a second set of experiments the action of OM-85 was investigated in the presence of bacterial (LPS) or pro-inflammatory (TNF-α) agonists. First, we focused on the effect of suboptimal concentrations of LPS (0.01 ng/ml) and OM-85 (10 µg/ml). As shown in Figure 3C, this combination strongly upregulated the release of CXCL8, CXCL1 and CXCL6 (not shown), at levels similar to those obtained with singular optimal concentrations of the two agonists (see Figure 2). Furthermore, the effect of an optimal OM-85 concentration (1 mg/ml) on CXCL8 production was also strongly increased by optimal (10 ng/ml) and suboptimal (1 ng/ml) TNF-α concentrations (Figure 3D).

### Activation of blood purified DC subsets by OM-85

**Figure 4:**
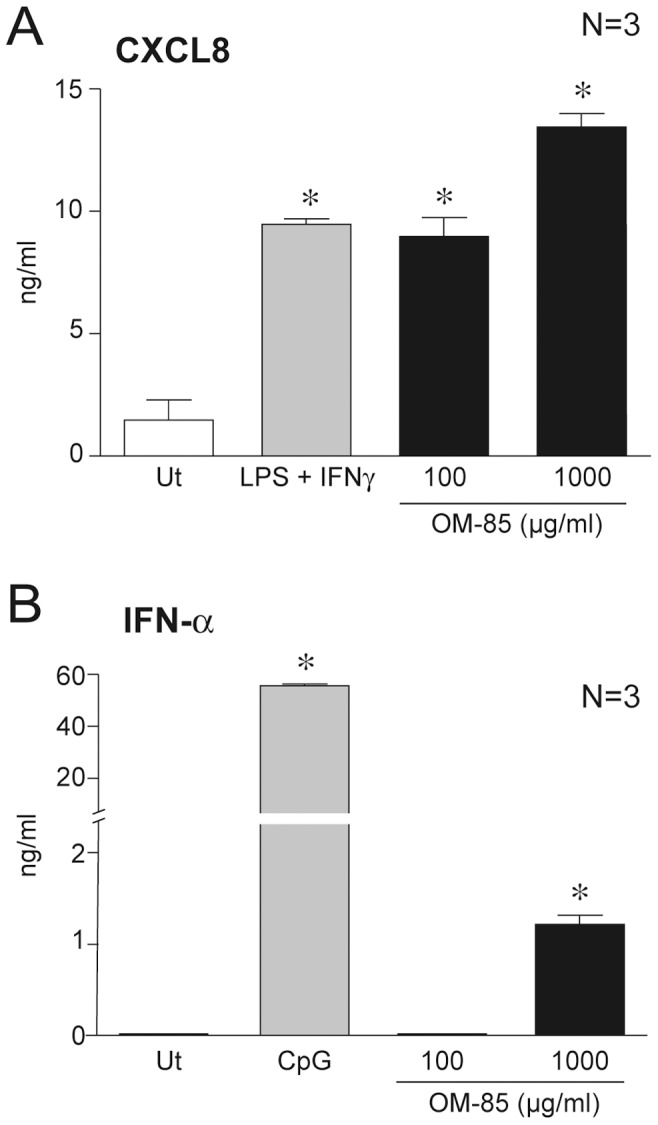
Activation of primary DC subsets by OM-85. **A**) MDC and **B**) PDC were isolated from buffy coats of three independent healthy donors and stimulated as indicated (10^6^/ml). After 24 hours, supernatants were collected and analyzed by ELISA. *P<0.05 by Dunnett's Multiple Comparison Test.

MDC and PDC were isolated from the blood of three independent donors and stimulated with 100 µg/ml or 1000 µg/ml OM-85 for 24 hours. LPS+IFNγ and CpG were respectively used as MDC and PDC prototypic activators. In agreement with the results obtained with MoDC, OM-85 induced the secretion of CXCL8 by MDC (Figure 4A) and IFNα by PDC (Figure 4B). Consistently with the results obtained with MoDC, OM-85 did not stimulate MDC to produce IL-12, IL-23 and CXCL10 (not shown).

### OM-85 activates cytokine production by immune cells from COPD patients

**Figure 5:**
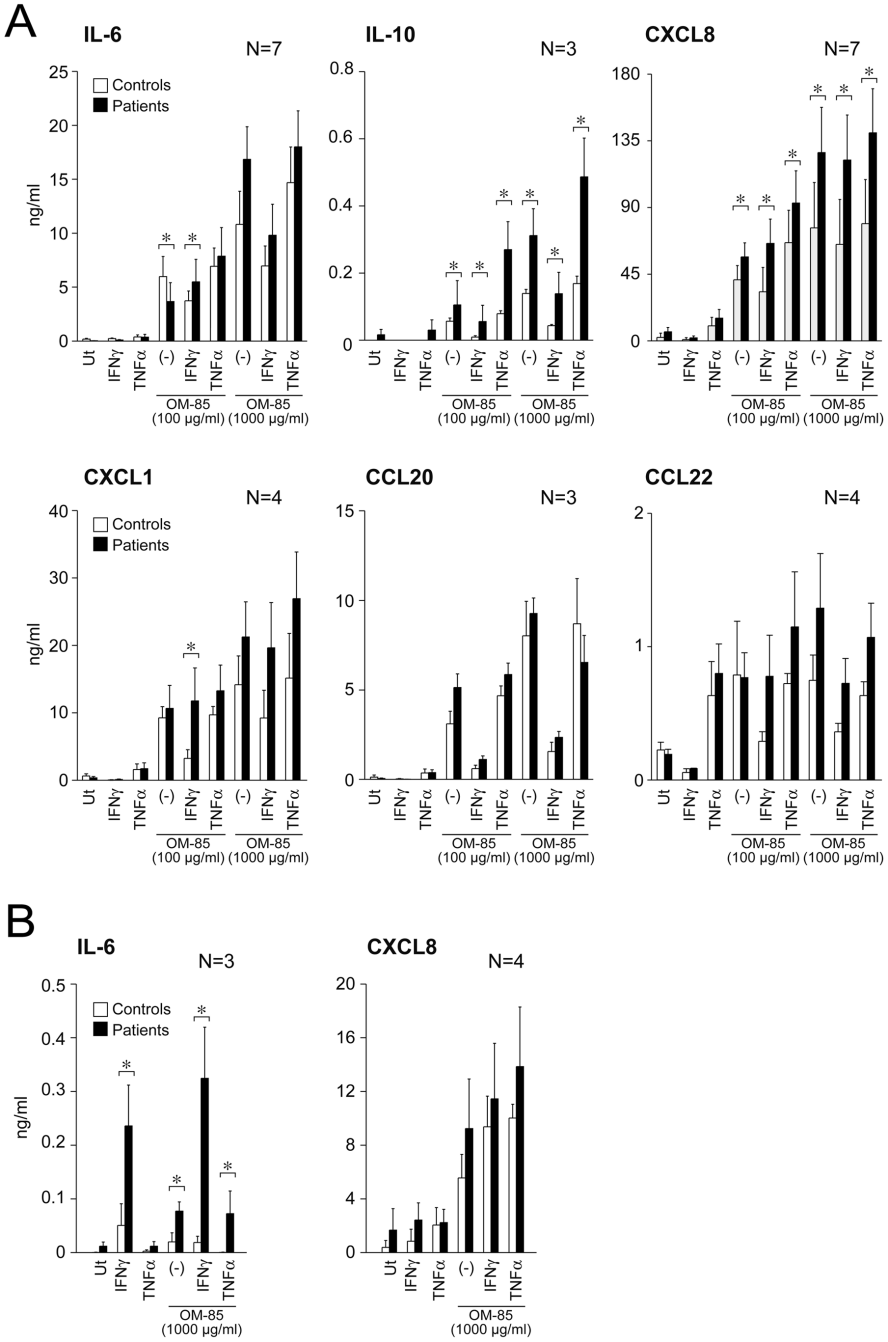
Activation of PBMC and MoDC from COPD patients and healthy subjects by OM-85. **A**) PBMC and **B**) MoDC (both 10^6^/ml) were stimulated as indicated in the presence or absence of 500 U/ml IFNγ or 100 ng/ml TNF-α. After 24 hours, supernatants were collected and analyzed by ELISA. Figure shows the results of healthy donors (open histograms) compared to COPD patients (black histograms). *P<0.05 by paired Student's *t* test.

OM-85 treatment is currently recommended in the prevention of acute exacerbations in COPD [5]–[6], a pathological condition often associated with alterations of the expression profile of PRR [16]. Thus, we set out to confirm previous results on cells from COPD patients. Given that we usually receive few ml of patients' blood which do not allow the purification of MDC, we choose to work with PBMC, which can be obtained in reasonable numbers from small amounts of blood and well represent the myeloid compartment of circulating cells. PBMC from COPD patients and healthy controls were stimulated with OM-85 and/or 500 U/ml IFNγ or 100 ng/ml TNF-α. As expected based on previous results, in response to OM-85 PBMC produced IL-6, IL-10, CXCL8, CXCL1, CCL20 and CCL22 (Figure 5A). In these experimental conditions, PBMC also produced low concentration of CXCL6 (0.32±0.09 ng/ml for 100 µg/ml OM-85; 0.3±0.11 ng/ml for 1000 µg/ml OM-85; n = 7, not shown).On the other hand, IL12p70, CXCL10 and BAFF release was below the assay detection limits.

In order to confirm at least part of these results on DC, that are the real focus of this work, we differentiated MoDC from blood of patients and healthy controls. Given the low number of MoDC recovered at the end of the differentiation process, we decided to concentrate our analysis on IL-6, IL-10 and CXCL8, for which a statistically higher production was detected in patients' PBMC. Figure 5B shows the results for IL-6 and CXCL8, while IL-10 was undetectable in these experimental conditions.

These experiments demonstrate that immune cells from COPD patients fully retain the capability to respond to OM-85. In addition, they show that COPD patients display a tendency to release higher levels of cytokines when stimulated with OM-85 alone or in combination with IFNγ or TNF-α, although this difference is statistically significant only in the case of IL-10 and CXCL8 for PBMC and IL-6 for MoDC (Figure 5).

## Discussion

Previous work reported that OM-85 has immunomodulating properties and may act on human DC by promoting DC maturation and T cell activation [11]. However, in order to elicit a full, pathogen-tailored immune response, DC must also secrete proper soluble mediators [17]. Here, we report that OM-85 induces the secretion of a defined set of cytokines by human DC through the NF-kB and MAPK pathways, which are activated downstream of the vast majority of PRR. By contrast, OM-85 did not activate the IRF pathway in these cells and experimental conditions, thus suggesting that TLR4, endosomal TLR and RLR may not be involved in OM-85 recognition and signaling. This conclusion contrasts with published data showing that activation of murine splenocytes by OM-85 was dramatically reduced in both TLR2−/− and TLR4−/− mice [8]. Nevertheless, species-specific differences may well explain such apparent discrepancy in TLR usage. The identification of the receptor/s triggered by OM-85 in human DC would be of great importance. We reasoned that, since OM-85 is a complex mixture of water soluble fractions from several bacterial strains, it is conceivable that cell activation does not rely on a single PRR, but depends instead on a number of PRR of different classes. Thus, we have started to investigate the activation of HEK293 cells transfected with single receptors. Preliminary data show that OM-85 triggers the production of CXCL8 in cells transfected with NOD1 and NOD2. In a different set of experiments, HEK293 cells were transfected with different TLR or NOD2 together with a reporter plasmid. Here, we detected some reporter activity in cells expressing TLR2 and NOD2. No signal could instead be activated by endosomal TLR, thus apparently supporting our conclusion based on missing IRF activation. However, these results are far from being conclusive because transfectants for a number of PRR are still missing. For example, since OM-85 contains peptidoglycans it is mandatory to investigate the role of C-type lectins and scavenger receptors. In addition, this strategy based on single transfectants may convey insufficient information because many PRR do work in heterodimers or even more intricate complexes, such as in the case of CD36, a coreceptor for TLR2/6 when sensing well defined PAMP. Thus, in order to depict the exact PRR pattern triggered by OM-85 in human DC, we aim at targeting in this cell type the most promising candidates identified by our screening experiments.

The activation of the NF-kB and MAPK pathways is known to lead to DC maturation and cytokine production. However, the cytokine panel induced by OM-85 is consistently different with respect to that induced by a prototypic, TLR4-dependent, DC activating stimulus, such as LPS. First, OM-85 was a very poor inducer of pro-inflammatory cytokines, exception made for the secretion of moderate levels of IL-6. In addition, OM-85, alone or in combination with IFNγ did not induce IL-12p70 and IL-23, two key TH1/TH17-polarizing cytokines. These results are in agreement with the lack of IL-12 production previously reported in a study performed using human lung fibroblasts stimulated with phytohaemagglutinin, but conflicts with a study performed with murine cells [8], strengthening the possibility that species-specific differences should be considered when analyzing the action of OM-85. In addition, OM-85 led to the production of BAFF and, in the presence of IFNγ+LPS, of IL-10. This panel of cytokine secretion proposes a role for OM-85 in the activation of the humoral arm, rather than the pro-inflammatory cellular arm, of the immune response. Indeed, both IL-6 and IL-10 are key mediators for B cell activation and survival [18]–[20] and BAFF is a key cytokine for the T cell-independent production of IgA by B cells [21]. This activation profile is conceivable with the induction of IgG and IgA production observed following in vivo administration of OM-85 in preclinical experimental models and in patients [22]–[23].

Given that OM-85 is recommended in the treatment of COPD, it is interesting to note that a similar cytokine profile was also observed using cells obtained from COPD patients, suggesting that the expression of the PRR involved in OM-85 recognition is not altered in this reactive pathological condition. The fact that cells from COPD patients tended to secrete more cytokines in response to stimulation was not unexpected since it is well known that inflammation is a central feature of COPD, which is characterized by increased transcription of pro-inflammatory proteins such as cytokines, chemokines, growth factors and enzymes [24]. In this context, the observed increase of IL-10 secretion by COPD cells might have a role in the control of excessive production of pro-inflammatory mediators and tissue damage and may represent a rationale for the usage of OM-85 in the prevention of acute exacerbations in COPD patients [5]–[6].

A second mechanism of action emerging from this study is the role of OM-85 in promoting the recruitment of immune effector cells through the release of chemokines. Indeed, DC exposed to OM-85 released increased levels of CCL2 and CCL3, two chemokines active on monocytes and NK cells, and CCL20 and CCL22, two chemotactic factors active at the epithelial surfaces [25]–[27]. Of particular interest is the ability of OM-85 to induce a PMN-skewed activation program through the production of CXCL1, CXCL6 and CXCL8, three chemokines that binds CXCR1 and CXCR2 on PMN. In agreement with these results, supernatants of OM-85-stimulated DC were active in inducing PMN migration in vitro. This finding supports a potential role of OM-85 in the activation of the first line of innate defense against bacterial infections. Of note, in the presence of sub-optimal concentrations of LPS (as low as 0.01 ng/ml) even uneffective concentrations of OM-85 became active in inducing the release of granulocyte-attracting chemokines and a similar action was observed in the presence of TNF-α, a master pro-inflammatory cytokine. Thus, at least for selected targets, OM-85 may be effective at lower concentrations when administered in the presence of a pre-existing pro-inflammatory background.

Finally, we report that OM-85 induced PDC to release low concentrations of IFNα, which represents the most important cytokine for the defense against viral infections. Thus, OM-85 may help to set up a basal antiviral state. This finding, while contrasting with the lack of IFN-I induction in MoDC, stresses the cell-specific mechanism of action and the importance to carefully evaluate the individual pathways of activation elicited by OM-85 in different target cells.

In conclusions, OM-85 induces a mild and well shaped human DC activation that may contribute to the generation of a “pre-alert state” resulting in an early protection towards incoming infections. This study provides the first description of the molecular basis for the activation of the immune system by OM-85. The evidence that DC exposed in vitro to OM-85 activate three distinct biological pathways to promote immune responses support the therapeutic effects observed in vivo and stresses the importance of a more detailed characterization of its mechanism of action in clinical settings.

## Supporting Information

Figure S1 **Lack of IRF pathway activation by OM-85 in MoDC.** **A**) Immature human MoDC were stimulated with 100 µg/ml OM-85 for 30, 60, 90 and 120 minutes. 100 ng/ml LPS was used as the positive control for IRF3 translocation, but it does not represent a positive control for IRF7 translocation. After cell lysis and protein fractionation, cytoplasmic (Cyto) and nuclear (Nuclei) extracts were blotted against IRF3 and IRF7. β-actin and Lamin A/C represent loading controls for cytoplasmic and nuclear proteins respectively. The image depicts results obtained in one representative donor out of eight. **B**) EMSA experiment showing lack of IRF-DNA binding activity by OM-85 in human moDC stimulated as in A). Signal specificity was assessed by competing each sample with a 125-fold excess unlabeled probe (lanes 2,4,6,8,10). The image depicts results obtained in one representative donor out of four. **C**) OM-85 induces no IRF-dependent gene transcription. Immature MoDC were stimulated with 100 µg/ml OM-85 (open circles) and 100 ng/ml LPS (black circles) for 2, 4, 8 and 24 hours. After RNA extraction, reverse transcription and DNAse I digestion, samples were amplified by Q-PCR using gene-specific primers. Results represent means+/−SE of three independent donors and are expressed as fold induction (FI) over unstimulated samples (0). **D**) OM-85 does not induce IFNβ1 secretion by MoDC. MoDC (10^6^/ml) were stimulated for 24 hours with LPS 100 ng/ml or OM-85 1000 µg/ml and supernatants analyzed by ELISA. *P<0.05 by paired Student's *t* test.
(TIF)
