# Supplementary figures and images for: Selective Activation of Human Dendritic Cells by OM-85 through a NF-kB and MAPK Dependent Pathway
Source: PLoS One. 2013 Dec 30;8(12):e82867. doi: 10.1371/journal.pone.0082867 (PMC3875422; doi:10.1371/journal.pone.0082867)

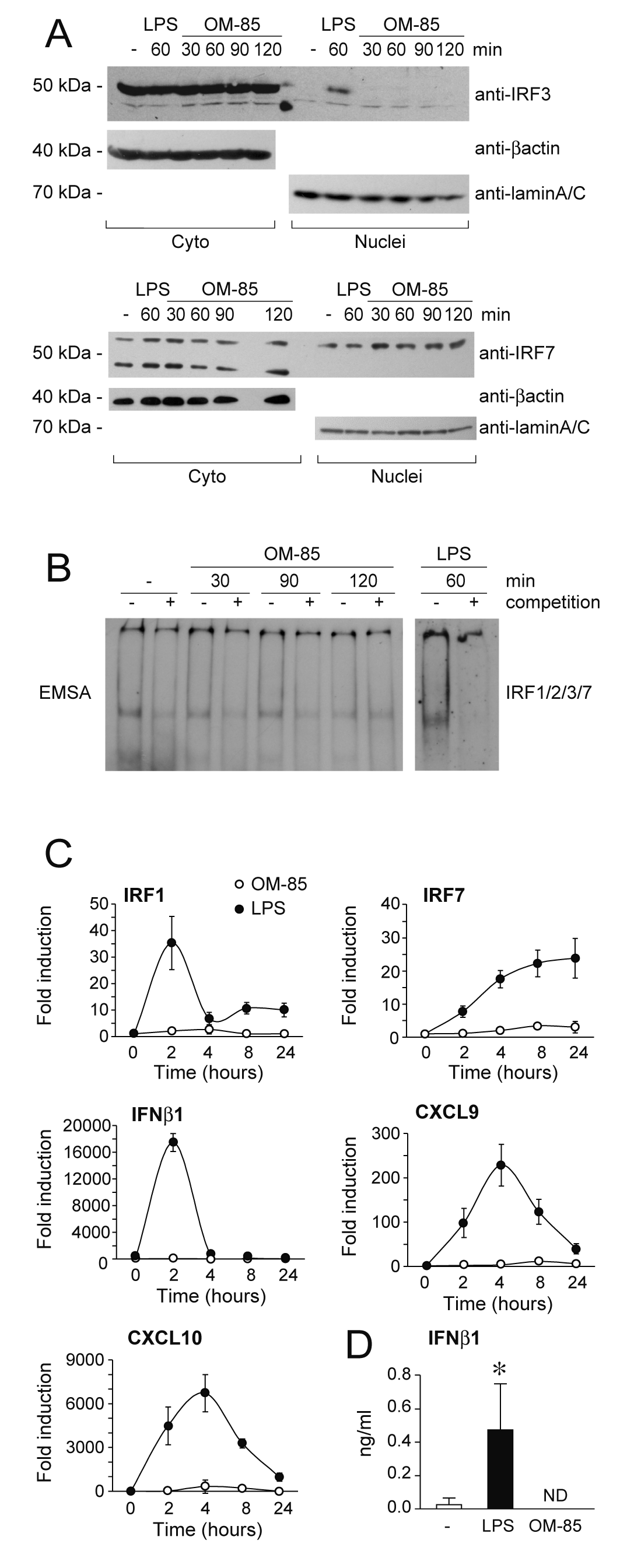

Supplement: Figure S1 — Lack of IRF pathway activation by OM-85 in MoDC. A) Immature human MoDC were stimulated with 100 µg/ml OM-85 for 30, 60, 90 and 120 minutes. 100 ng/ml LPS was used as the positive control for IRF3 translocation, but it does not represent a positive control for IRF7 translocation. After cell lysis and protein fractionation, cytoplasmic (Cyto) and nuclear (Nuclei) extracts were blotted against IRF3 and IRF7. β-actin and Lamin A/C represent loading controls for cytoplasmic and nuclear proteins respectively. The image depicts results obtained in one representative donor out of eight. B) EMSA experiment showing lack of IRF-DNA binding activity by OM-85 in human moDC stimulated as in A). Signal specificity was assessed by competing each sample with a 125-fold excess unlabeled probe (lanes 2,4,6,8,10). The image depicts results obtained in one representative donor out of four. C) OM-85 induces no IRF-dependent gene transcription. Immature MoDC were stimulated with 100 µg/ml OM-85 (open circles) and 100 ng/ml LPS (black circles) for 2, 4, 8 and 24 hours. After RNA extraction, reverse transcription and DNAse I digestion, samples were amplified by Q-PCR using gene-specific primers. Results represent means+/−SE of three independent donors and are expressed as fold induction (FI) over unstimulated samples (0). D) OM-85 does not induce IFNβ1 secretion by MoDC. MoDC (106/ml) were stimulated for 24 hours with LPS 100 ng/ml or OM-85 1000 µg/ml and supernatants analyzed by ELISA. *P<0.05 by paired Student's t test. (TIF) [file pone.0082867.s001.tif]
